# Computational Investigation of the Effect of Lipid Membranes on Ion Permeation in Gramicidin A

**DOI:** 10.3390/membranes6010020

**Published:** 2016-03-18

**Authors:** Jeffry Setiadi, Serdar Kuyucak

**Affiliations:** School of Physics, University of Sydney, Sydney NSW 2006, Australia; jeffry.setiadi@sydney.edu.au

**Keywords:** gramicidin A, ion permeation, molecular dynamics, potential of mean force, ceramides

## Abstract

Membrane proteins are embedded in a lipid bilayer and interact with the lipid molecules in subtle ways. This can be studied experimentally by examining the effect of different lipid bilayers on the function of membrane proteins. Understanding the causes of the functional effects of lipids is difficult to dissect experimentally but more amenable to a computational approach. Here we perform molecular dynamics simulations and free energy calculations to study the effect of two lipid types (POPC and NODS) on the conductance of the gramicidin A (gA) channel. A larger energy barrier is found for the K${}^{+}$ potential of mean force in gA embedded in POPC compared to that in NODS, which is consistent with the enhanced experimental conductance of cations in gA embedded in NODS. Further analysis of the contributions to the potential energy of K${}^{+}$ reveals that gA and water molecules in gA make similar contributions in both bilayers but there are significant differences between the two bilayers when the lipid molecules and interfacial waters are considered. It is shown that the stronger dipole moments of the POPC head groups create a thicker layer of interfacial waters with better orientation, which ultimately is responsible for the larger energy barrier in the K${}^{+}$ PMF in POPC.

## 1. Introduction

Gramicidin A (gA) is an antibiotic peptide that disrupts bacteria by allowing unimpeded flow of cations into the plasma membrane. This small hydrophobic peptide is composed of two identical helical subunits with 16 residues on each. The two subunits form a stable compound only inside a lipid bilayer environment. When stable, the gA dimer forms a narrow cylindrical hole across the bilayer, through which water and monovalent cations can permeate near diffusion rates. The channel structure was determined using solution [1] and solid-state NMR [2], and consists of a single-stranded, right-handed *β*-helical dimer. Each subunit is made up of formyl-VGALAVVVWLWLWLW-ethanolamine (underlined residues indicate D-amino acids and L-amino acids otherwise) [3]. The alternating L-D amino acid sequence allows the peptide to fold into a helix with the side chains aligned on the exterior of the helix [4]. There are two high-resolution structures available, PDB:1MAG [5] and PDB:1JNO [6]. These two structures have been used in a large number of computational studies, investigating the ion permeation properties of gA [7,8,9,10,11]. Due to its simple structure, gA has often been used as a model for membrane proteins. For example, it was used as a prototype ion channel model long before the first potassium ion channel protein was crystallized [12,13]. It has also been used as a testing model for developing and validating computational methods from continuum theories to ab initio molecular dynamics (MD) [14,15,16,17,18].

Biological membranes play an important role in cell biology. They consist of lipid molecules organized in a bilayer formation, leaving a ∼30 Å hydrophobic layer between the intracellular and extracellular environment of cells [19]. Membrane proteins embedded in lipid bilayers may have their functions modulated by the surrounding environment as shown in some studies [20,21,22,23]. Because gA is a small protein, the effects of lipids on its function is expected to be greater than those of larger membrane proteins. Hence, gA is a good candidate to investigate the effects of lipid environment on protein function.

The effect of membrane environment on gA function have been investigated in several studies previously [24,25,26,27,28]. Of particular interest is the work of Cukierman and co-workers, where gA is embedded in four different lipid environments; monoglycerides, ceramides, sphingolipids and phospholipids [24]. The experiments showed a markedly decreased ion conductance for gA in a phospholipid environment compared to ceramides. On the other hand, the proton conductance exhibits the opposite behavior with phospholipids enhancing conductance [25]. Enhancement in proton transport is attributed to the orientation of water molecules at the membrane-water interface due to the polar phosphate head groups [26]. Proton transport involves jumps between hydronium and water molecules connected through hydrogen-bonds [27]. Alignment of water molecules at the interface, due to the membrane dipole potential [28], reduces entropy thereby decreasing the energy barrier for proton transport. Ion transport, however, proceeds through hydrodynamic diffusion. The role of the phosphate head groups and the aligned water molecules at the lipid interface in ion transport across gA is still not clear.

**Figure 1:**
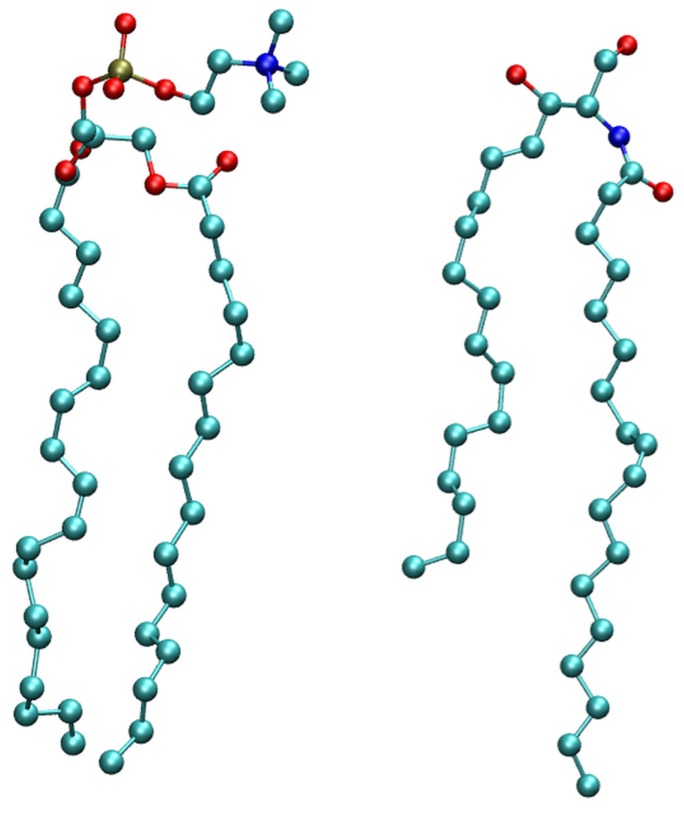
Molecular structure of phospholipids (POPC) (**left**) and ceramides (NODS) (**right**) lipid molecules. Hydrogen atoms are not shown for clarity.

In this article, we attempt to explain the origin of the effects of lipid environment on ion conductance of gA channel. We test two different lipid molecules, comparing phospholipids (POPC) and ceramides (NODS) (Figure 1). This is accomplished using MD simulations and free energy calculations. Our results show that ion conductance is indeed enhanced by ceramide lipids consistent with the experimental observations. The reduction in conductance for phospholipids comes from two sources, the phosphate head groups and the orientation of water molecules in the interface layer.

## 2. Results and Discussion

### 2.1. Membrane Bilayer

**Figure 2:**
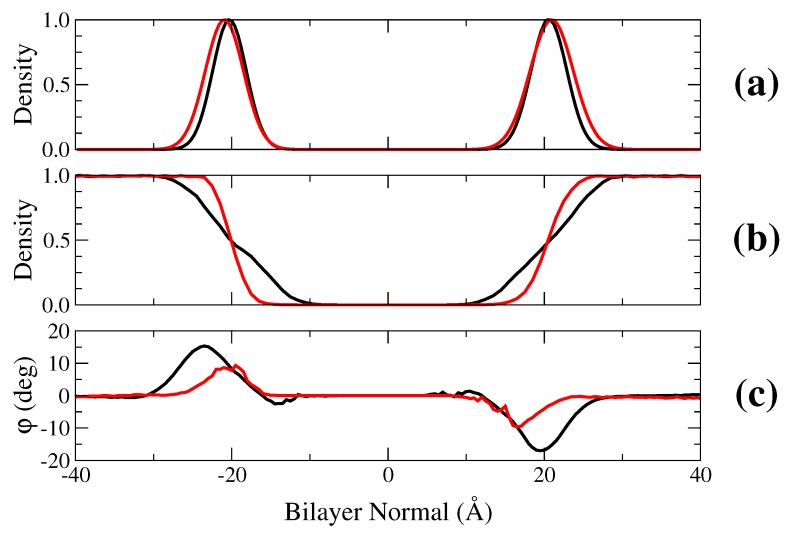
Analysis of the pure bilayer systems: (**a**) mass density of head groups; (**b**) water number density; and (**c**) average orientation of water molecules with respect to the bilayer plane (the angle between the dipole vector and the *x-y* plane). Black and red lines represent systems with POPC and NODS lipids, respectively. Densities are calculated using the density profile extension in VMD [34].

Before investigating ion conductance in gA we studied the behavior of the lipid bilayers with POPC and NODS. The molecular structures of POPC and NODS are shown in Figure 1. For the purpose of investigating pure bilayers, we used a simulation system consisting of 64 lipid molecules per leaflet. To compare the thickness, we calculated the mass density of the head groups for each lipid type and plotted the densities in Figure 2. For POPC, we chose the phosphorus atom as the reference, and the oxygen on the hydroxyl group in NODS. From 4 ns of MD simulations, we sampled the location of the head groups along the bilayer normal (*z*-axis). From the maximums, it can be seen that the oxygen atoms are located slightly further away from the bilayer center compared to the phosphorus atoms (21.0 Å and 20.3 Å, respectively). Thus, the average bilayer thickness is about 41 Å and 42 Å for POPC and NODS, respectively, in agreement with experiments and simulations [29,30]. It has been shown that the asymmetric hydrocarbon chains of ceramide results in a thicker bilayer formation [31]. We observed a larger difference in the horizontal densities (*i.e.*, area per lipid) of POPC and NODS lipids. For POPC we found an area of ~60 Å${}^{2}$ per lipid in good agreement with the experimental values [32]. For NODS bilayer, the area per lipid we obtained is ~46 Å${}^{2}$, again very similar to the experimental value [33]. These give confidence to the force field parameters used in the simulations. The large difference between POPC and NODS densities is simply due to the larger head group in POPC. We also plotted the water density along the bilayer normal in Figure 2. The water density profiles show a thicker layer of water molecules at the interface for POPC than NODS. This behavior is a direct result of the tighter packing of NODS molecules in the bilayer formation.

To further analyze the previous observation, we plotted the average orientation of water molecules as a function of the bilayer normal. The orientation is defined as the angle between the dipole moment of water molecules and the bilayer plane. The angles are averaged over a 2 ns trajectory collected in 0.5 Å bins. The peak of the profiles shown in Figure 2 is roughly 20${}^{\circ }$ and 10${}^{\circ }$ for POPC and NODS, respectively. Qin *et al.* obtained a peak close to 30${}^{\circ }$ for a DiPhPC bilayer [26]. The difference between our results and ref. [26] for the PC head groups can be attributed to polarization effects that is included semi-empirically in the multistate empirical valence bond model [35]. However, even without polarization, our results clearly show that the PC head groups orient water molecules over a wider range than for ceramide. Thus the larger head groups in POPC not only increases the area per lipid but also results in a greater dipole potential. It is interesting to compare the water orientation with GMO bilayers [26], where water molecules exhibit close to random orientation on average at the interface. Experimental measurements of cesium conductance through gA embedded in GMO bilayers show very similar behavior to NODS at concentrations greater than 250 mM [24]. The orientation of water molecules at the interface may not be the only factor contributing to the enhanced conductance.

### 2.2. gA Embedded in Membrane Bilayer

**Figure 3:**
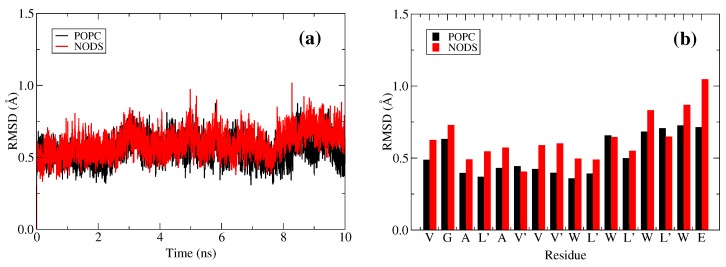
RMSD of gA embedded in POPC and NODS lipids: (**a**) total and (**b**) average per residue. Error bars are not shown for RMSD per residue for clarity.

After embedding the gA in bilayers we have simulated the equilibrated system for 10 ns with only water molecules inside. The resulting RMSD, calculated by excluding the ethanolamine groups, is shown in Figure 3. The RMSD fluctuates around 0.5–0.8 Å and there are some slight changes in RMSD over the trajectory, but there are no perceptible differences between the RMSDs of gA embedded in POPC and NODS. When we include ethanolamine in the calculations, the RMSD time series fluctuates closer to 1 Å. To show this effect quantitatively, we plot the average RMSD per residue including the ethanolamine, which is also shown in Figure 3. For POPC, all the residues have RMSD values less than 0.75 Å. In NODS, however, the residues near the entrance of the channel fluctuate more than POPC because the gA side chains have much weaker interactions with the NODS head groups compared to those of POPC. In particular, the ethanolamine group swings instantaneously and this is also observed in MD simulations for gA with other bilayers [36]. The swing motion perturbs the neighboring residues thereby explaining the slight turns in the RMSD time series even without ethanolamine. The swing motion of ethanolamine is due to loss of hydrogen-bonds [37], and we circumvent this problem by sampling the systems longer. Despite these minor differences, gA is essentially stable inside the hydrophobic environment of both lipids. With gA embedded inside the membrane, we find that the bilayer thickness of the lipid molecules in the first shell decreases appreciably as observed by Kim *et al.* previously [36]. The bilayer thickness is recovered in the second lipid shell and hence only lipids in the first shell are affected. Because we have embedded gA in 20 lipid molecules per leaflet configured in a hexagonal cell, we report only the first shell results here. The bilayer thickness, as defined in the previous subsection, decreases from 41 to 31 Å for POPC, and from 42 to 37 Å for NODS. The larger decrease in the thickness of a POPC bilayer is again related to the much stronger coupling of the POPC head groups with the gA side chains compared to those of NODS.

**Figure 4:**
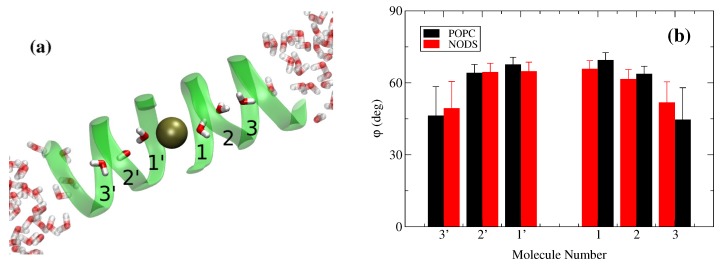
Water molecules inside the channel with K${}^{+}$ located at the center of gA. (**a**) Structure showing K${}^{+}$ positioned in the center of gA with aligned water molecules (lipids not shown); and (**b**) average orientation of water molecules. The orientation is defined as the angle between the dipole vector and the *x-y*-plane. Labels for water molecules are shown on the diagram.

In the previous trajectory of the gA system with water molecules, we have replaced a water molecule near the center of gA with a K${}^{+}$ ion. We have applied a harmonic restraint to keep the K${}^{+}$ ion at the center and sampled the systems for a further 4 ns (the ethanolamine group is stable at this point). To gain further insights into the potential contributors to the ion conductance, we have analyzed the behavior of water molecules inside the channel. When the K${}^{+}$ ion is positioned at the center of gA, there are three water molecules aligned on either side of the ion inside the channel. Similar to the results in Figure 2, we have calculated the average orientation of the six water molecules in gA with respect to the bilayer plane (Figure 4). As expected, the alignment of the water dipoles with the channel axis, and hence the strength of the interaction between the ion and water molecules, decreases as a function of the distance from the ion. We note that the water dipoles are not aligned closer to 90${}^{\circ }$ with the plane because they make hydrogen-bonds with the carbonyl groups of gA. Taking into account the statistical fluctuations, the interaction between the K${}^{+}$ ion and water molecules inside the channel are very similar for the POPC and NODS bilayers.

### 2.3. Potential of Mean Force of K${}^{+}$ in gA

**Figure 5:**
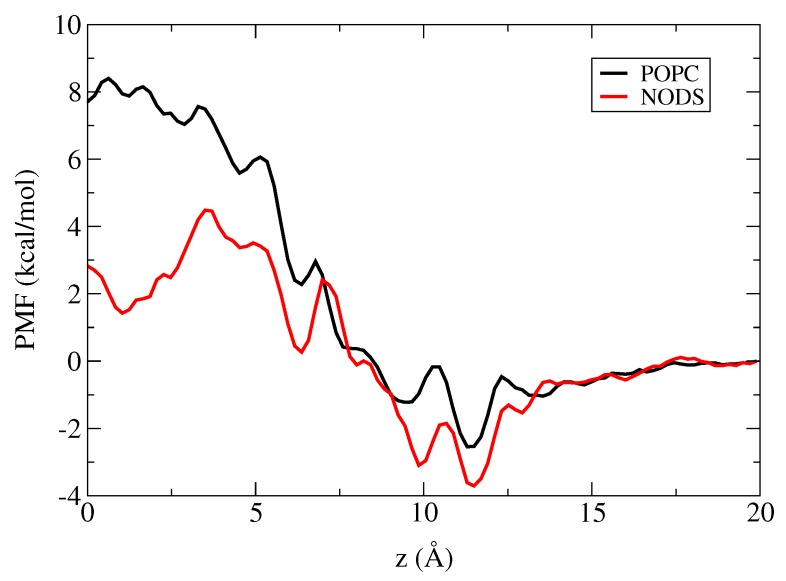
Potential of mean force (PMF) profiles of a K${}^{+}$ ion along the gA channel axis, *z*.

We have performed umbrella sampling simulations to determine the PMFs of a K${}^{+}$ ion along the central axis of gA embedded in the POPC and NODS bilayers. Details of the umbrella sampling simulations are described in the Methods section. Each umbrella window is simulated for 3 ns. Using the stability of the ethanolamine group and the convergence of the PMFs as criteria, the first 1 ns of data are discarded for equilibration and the PMFs are constructed from the final 2 ns of production data. The PMFs obtained with the POPC and NODS bilayers are shown in Figure 5. The PMFs exhibit similar behavior from the bulk region up to ∼7.5 Å inside the channel but start diverging from there to the center of gA. There is also some difference in the binding free energies at the binding pocket (∼11.3 Å). For the POPC bilayer, we obtain a well depth of 2.5 kcal/mol at the binding site with respect to bulk. In previous PMF calculations for a K${}^{+}$ ion, well depths in the range of 2–3 kcal/mol were obtained for PC bilayers [8,9,10,11]. As the accuracy of the PMF calculations is about 1 kcal/mol, the present result for the well depth is consistent with those earlier results. In the case of NODS, the well depth at the binding site is 3.7 kcal/mol, which is 1.2 kcal/mol deeper compared to the POPC bilayer. Thus we predict an eight-fold difference between the binding constants of K${}^{+}$ ions for gA in POPC vs NODS bilayers, which can be easily distinguished in experiments.

The two PMFs start diverging around 7.5 Å, and the difference becomes quite substantial near the center of gA. The energy barrier measured from the binding site to the peak in the PMF is 10.9 kcal/mol for POPC and 8.2 kcal/mol for NODS. The barrier value for POPC is again in agreement with the previous PMF calculations in gA [8,9,10,11]. The lower energy barrier observed in the NODS bilayer compared to that in the POPC bilayer is consistent with the experimental data which shows a four-fold increase in cation conductance of gA embedded in NODS compared to POPC [24]. We stress that polarization plays a significant role in ion permeation across the gA channel, hence MD simulations with a non-polarizable force field provide only qualitative results.

**Figure 6:**
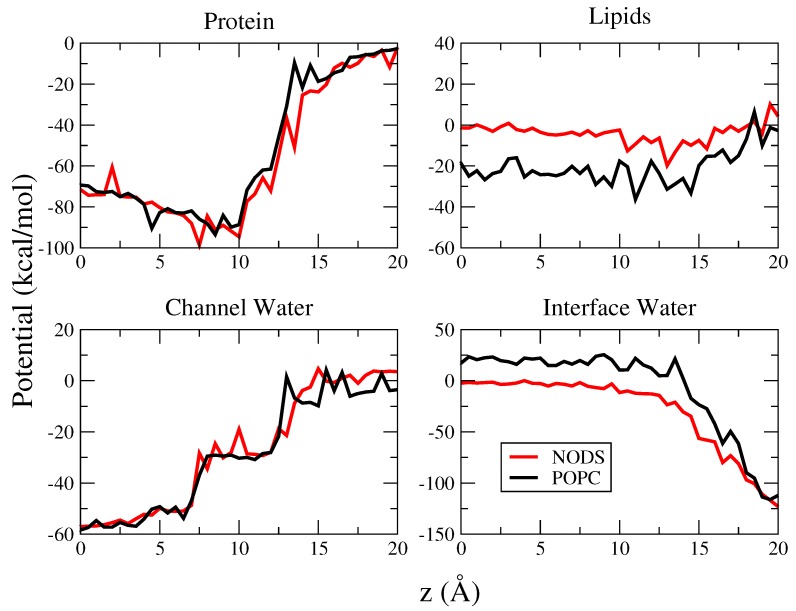
Potential energy acting on K${}^{+}$ ion as a function ion positions in the gA channel. Interactions calculated include protein, lipid molecules, channel water and interfacial water. Red and black line represents POPC and NODS lipid molecules.

It is of interest to find out how the change in the lipid bilayer affects the ion PMFs. To determine the cause for this difference in the PMFs, we calculate the average potential energy acting on the K${}^{+}$ ion from four different components of the system individually. The potential energy is calculated from the umbrella sampling trajectory data at 0.5 Å intervals. The four components we chose to calculate are the protein, the lipid molecules, and the water molecules inside the channel and at the lipid interface. As shown in Figure 2 and Figure 4, these water molecules exhibit alignment with the channel axis or the bilayer normal, hence will contribute to the potential energy of the ion. The channel water molecules are defined as the water molecules inside the region [−10, 10] Å and interfacial water is defined by the range of molecules oriented by the lipid head groups as shown in Figure 2. The K${}^{+}$ potential energies due to these four components are plotted as a function of gA channel axis in Figure 6. We note that the potential energy does not include entropy effects, hence we focus on qualitative rather than quantitative results.

We first consider the potential energy of the K${}^{+}$ ion due to gA. As expected, this potential energy approaches zero as the ion moves further into the bulk region. As the ion moves closer to the binding site the potential energy decreases to a minimum and then slowly increases as it reaches the center. This trend is consistent with Allen *et al.* [8], but the values we calculate are of a different scale because we consider the potential energy rather than integrating the mean force. For both POPC and NODS the potential energy profiles are very similar as evident from Figure 6. This result indicates that the lipid environment does not directly affect the interaction between gA and the ion inside the channel. Kim *et al.* investigated behavior of gA in different phospholipid environments and observed variations in the dynamics of gA [36]. From our results we conjecture that although the lipid environment can perturb gA dynamics due to hydrophobic mismatch, these perturbations may not be significant enough to change the nature of the interaction between gA and K${}^{+}$.

Considering next the potential energy of the K${}^{+}$ ion due to the lipid molecules, we see a noticeable difference between the POPC and NODS bilayers Figure 6. The potential energy due to NODS remains close to zero throughout, with a slight change from negative near the binding site to positive as the ion moves towards the bulk. This is mainly due to the interaction between K${}^{+}$ and the NODS head groups, which have small dipole moments and a short range. For POPC, however, the potential energy is quite substantial, which is the result of the strong dipole potential of the PC head groups. The potential energy remains negative within gA and approaches zero as the ion moves towards the bulk as expected. On average, the difference in energy between the two lipid bilayers is ∼20 kcal/mol within gA, and it favors POPC relative to NODS. This is clearly in the opposite direction to the PMF profiles, where the NODS PMF is lower than the POPC PMF (Figure 5). Thus other contributions to the K${}^{+}$ potential energy are needed to explain the difference in the PMFs.

The other components that could influence the potential energy of the K${}^{+}$ ion in gA are water molecules. Water molecules can be separated into three groups based on their orientation in the system. Firstly, water molecules with random orientation are located in the bulk region, where interactions with protein and membrane are negligible. Next, as discussed earlier, a layer of water molecules tend to orient away from the bilayer due to membrane dipole potential. Finally, the single-file water molecules inside the channel align their dipole moments with the electric field of the ion, which is modulated by the formation of hydrogen-bonds with the carbonyl oxygens of gA. Here we are interested only in water molecules with specific orientation, and hence consider only the potential energy due the channel and interfacial water molecules.

We start with the interaction of the channel waters with the K${}^{+}$ ion. We have stated earlier that the behavior of the dipole moments of water molecules in gA, in the presence of a K${}^{+}$ ion at the center, is approximately the same in the NODS and POPC bilayers (Figure 3). This behavior is seen to be maintained for other positions of the K${}^{+}$ ion in gA—the potential energies of the K${}^{+}$ ion due to the channel waters are seen to overlap well for the two lipid molecules (Figure 6). The small differences observed when the ion is outside gA is likely arise from the flipping of the dipole moments of water molecules. This verifies that the water molecules inside the channel do not directly contribute to the attenuation and enhancement of ion conductance in gA embedded in POPC and NODS bilayers, respectively. It is interesting to note the trend of the interaction inside the channel. There are three different stages in the range [0, 20] Å where the interaction changes. Starting from 20 Å, the potential energy is on average close to zero as expected. As the ion is moved closer to the binding site, the potential decreases to -30 kcal/mol. This sudden change in potential is the result of one water molecule being pushed out of the channel on the other side to accommodate the K${}^{+}$ ion. From the binding site to 7.5 Å, the potential energy remains steady. However, as the ion is pushed further to the center, there is a second dip in the potential energy, bringing it down to about -50 kcal/mol. We have observed that this results from the reorientation of water molecules inside the channel. The potential energy comes to a minimum at the center with -60 kcal/mol, where the dipole moments of three water molecules are aligned in the direction of the ion’s electric field on either side of the channel in a symmetric configuration. The potential energy profile demonstrates the stages that take place as the ion moves through gA, which cannot be traced from the PMF alone.

Lastly, we consider the interaction of K${}^{+}$ with the interfacial water molecules, which exhibits a significant difference in the potential energy of K${}^{+}$ with the NODS and POPC bilayers. For NODS, the potential energy is zero at the center and decreases to about -10 kcal/mol at the binding site. For POPC on the other hand, the potential energy is around +25 kcal/mol in gA and only starts to decrease around the binding site. As the ion is moved further away from gA, the potential energy of both systems converge to the same level. We note that the potential due to the interfacial waters will be strongly screened by bulk water and the free energy of K${}^{+}$ will vanish in bulk as seen in the K${}^{+}$ PMF (Figure 5). To understand the difference between the POPC and NODS results, we refer to Figure 2 which shows that there is a thicker layer of water molecules in POPC, better oriented by the PC head groups with larger dipole moments. This results in a much stronger dipole potential due to the interfacial waters when gA embedded in a POPC bilayer compared to that of NODS. Comparing the lipid contribution to the potential energy of K${}^{+}$ with that of the interfacial waters (Figure 6), it is seen that the latter contribution more than compensates the former in POPC, resulting in a positive potential energy in gA. In contrast, the sum of the two contributions remain near zero but slightly negative in NODS. Thus our results indicate that the larger energy barrier in the POPC PMF relative to the NODS PMF is most likely due to the better orientation of the interfacial water molecules by the stronger dipole potential of the POPC head groups.

## 3. Materials and Methods

### 3.1. Model System

The two lipid molecules considered are 1-palmitoyl-2-oleoyl-sn-glycero-3-phosphatidylcholine (POPC) and N-oleoyl- D-erythrosphingosine or ceramide C18 (NODS). The structure of both molecules are generated using the Avogadro software [38]. For POPC, we use the standard topology and parameters available in CHARMM36 [39]. The topology for NODS is generated from the ParamChem server [40,41] and is derived from sphingomyelin parameters available in CHARMM36 [42]. One dihedral parameter with a penalty higher than 10 is optimized using the force-field toolkit (FFTK) [43] available in VMD [44]. The target data for optimization are generated using Gaussian 09 [45].

The 1NJO structure [6] is used for the gA dimer embedded in a lipid bilayer. The gA dimer is placed in a hexagonal cell with 20 lipid molecules placed around the peptide per layer. We solvate the gA–membrane complex with TIP3P water molecules and neutralize the system with 0.15 mol/L of KCl. The system is equilibrated in two stages to ensure the stability of the peptide-membrane complex. First, the gA atoms are fixed and the cell is allowed to fluctuate isotropically until the correct lipid and water densities are obtained. The cell in the *x*-*y* plane is then fixed and only fluctuations in the z direction are allowed. In the second stage, the gA atoms are gradually relaxed over 3 ns of equilibration. The system is further equilibrated without any restraints for 10 ns. We have performed the production runs without any restraints on the gA atoms because the flexibility of the protein affects ion permeation as demonstrated in a previous work [16]. To keep the protein stable inside the bilayer, we apply harmonic restraints on the orientation and position of the center of mass of the protein. The restraint on the center of mass does not affect the internal degrees of freedom of the protein.

### 3.2. PMF Calculations

We calculate the potential of mean force (PMF) of potassium ion along the gA channel using the equilibrium umbrella sampling simulations [46] with the weighted histogram analysis method [47]. We use the same protocols as in an earlier work [11] for configuring the calculations, which are briefly described here. The reaction coordinate for the ion is its distance from the gA center along the channel axis. We perform the PMF calculations in the range [0, 20] Å, assuming symmetric behavior between the two monomers. For umbrella sampling calculations, we use a total of 41 windows at 0.5 Å intervals. The umbrella windows are generated via steered MD simulations, starting from the equilibrated system with the K${}^{+}$ ion at the center of gA. We apply a harmonic potential with a force constant of 10 kcal/mol, which is reduced to 7 kcal/mol outside the pore (z>15 Å) to improve sampling between windows. In the bulk region, a harmonic potential of 1 kcal/mol is applied in the radial direction to prevent the ion drifting away from the central axis. Each window is run for 3 ns with 1 ns for equilibration, giving a total simulation time of 123 ns.

### 3.3. MD Simulations

All MD simulations in this work are performed with the NAMD package (version 2.10) [48] with the CHARMM force field [39]. We employ the NPT ensemble and kept the simulation temperature constant at 300 K using the Langevin thermostat with a damping factor of 1 ps${}^{-1}$. The pressure is kept constant at 1 atm using the Langevin piston method with a damping factor of 50 ps${}^{-1}$ [49]. Periodic boundary conditions are used and electrostatic interactions are calculated using the particle-mesh Ewald method [50] without truncation. Non-bonded interactions are truncated at 12 Å and replaced with a smooth switching function starting from 10 Å. In all simulations a time step of 2 fs is employed for the integrator.

## 4. Conclusions

Our objective in this work is to understand the cause of the enhancement of ion conductance in gA embedded in a NODS bilayer compared to that of POPC. For pure bilayer systems, we have observed different lipid–water interactions with the result that water molecules tend to be more structured in phospholipids than ceramide. This behavior was also demonstrated in comparisons of phospholipids with monoglycerides [26]. Our simulations of the gA system show that the peptide behaves in a similar fashion when it is embedded in both lipid environments. However, the PMF of a K${}^{+}$ ion in gA embedded in POPC results in a distinctly larger energy barrier compared to that of NODS, which is consistent with the observed enhancement of the conductance in NODS [24]. To understand the origin of this enhancement, we have analyzed the different contributions to the potential energy of the K${}^{+}$ ion from gA, lipid molecules and water molecules within gA and at the lipid interface. We find that the interaction of gA and water molecules inside gA with K${}^{+}$ are virtually identical for both lipid molecules. But there are substantial differences between the POPC and NODS bilayers when we consider the interaction of lipid molecules and interfacial waters with K${}^{+}$. In NODS, the weak dipole moments of the head groups result in a loosely structured interfacial waters, and the contribution from either group to the ion’s potential energy is very small. In POPC, the strong dipole moments of the head groups give rise to a thicker layer of interfacial waters with better orientation. Thus, both groups make substantial contribution to the ion’s potential energy, but the positive contribution from the interfacial waters overcomes the negative one from lipids. This difference is consistent with the higher energy barrier found in the K${}^{+}$ PMF in POPC compared to the K${}^{+}$ PMF in NODS.

## Abbreviations

The following abbreviations are used in this manuscript:

gA: gramicidin A
MD: molecular dynamics
NODS: ceramide C18, N-oleoyl-D-erythro-sphingosine
PC: phosphatidylcholine
POPC: phospholipid, 1-palmitoyl-2-oleoyl-sn-glycero-3-phosphatidylcholine
PMF: potential of mean force
